# No Effect of Isolated Anthocyanins from Bilberry Fruit and Black Rice on LDL Cholesterol or other Biomarkers of Cardiovascular Disease in Adults with Elevated Cholesterol: A Randomized, Placebo‐Controlled, Cross‐Over Trial

**DOI:** 10.1002/mnfr.202101157

**Published:** 2022-04-26

**Authors:** Hassan Aboufarrag, Wendy J. Hollands, Jasmine Percival, Mark Philo, George M. Savva, Paul A. Kroon

**Affiliations:** ^1^ Quadram Institute Bioscience Norwich Research Park Norwich NR4 7UQ UK; ^2^ Food Science and Technology Department Faculty of Agriculture Alexandria University Alexandria 23511 Egypt

**Keywords:** flavonoids, glucose control, low density lipoprotein, paraoxonase‐1, *Vaccinium myrtillus*

## Abstract

**Scope:**

Some dietary interventions with berry fruits, berry fruit extracts, and purified anthocyanins have been reported to beneficially alter lipoprotein profiles in hyperlipidemic participants. The major anthocyanins in human diets are glycosides of cyanidin and delphinidin, and structure can influence both absorption and bioactivity. The aim of this study is to determine the effects of two major types of anthocyanins on low‐density lipoprotein cholesterol and other cardiometabolic markers for cardiovascular disease (CVD) risk in hyperlipidemic individuals.

**Methods and results:**

Fifty‐two hyperlipidemic participants complete this randomized, placebo‐controlled, double‐blind, three arm crossover trial. Participants ingest capsules containing 320 mg of anthocyanins (bilberry trihydroxy‐type or black rice dihydroxy‐type) or placebo once daily for 28 days. Biomarkers of CVD risk are measured before and after the intervention period. Compared to the placebo, neither anthocyanin treatment significantly (*p* < 0.05) changes circulating levels of lipoproteins (total‐/high‐density lipoprotein (HDL)‐/low‐density lipoprotein (LDL)‐cholesterol, triglycerides, Apolipoprotein B (ApoB)), biomarkers of glycemic control (fasting glucose, fructosamine), biomarkers of HDL function (ApoA1, HDL3, paraoxonase‐1 (PON1) arylesterase, and lactonase activities), or plasma bile acids.

**Conclusions:**

These data do not support the notion that regular consumption of anthocyanins beneficially affects glycemic control or lipoprotein profiles or functions. It is possible the no effect observation is due to the relatively short duration of treatments.

## Graphical abstract

Daily consumption for 28 days of isolated anthocyanins from black rice and bilberry does not affect biomarkers of lipoprotein metabolism, lipoprotein quality, or glucose control.

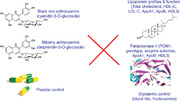

## 1 Introduction

Cardiovascular disease (CVD) is the leading cause of death globally.^[^ ^1^ ^]^ Elevated levels of low‐density lipoprotein cholesterol (LDL‐C) and triglycerides contribute significantly to the development of atherosclerosis,^[^ ^2^, ^3^ ^]^ an underlying pathophysiological cause of CVD. Elevated levels of circulating high‐density lipoprotein cholesterol (HDL‐C) on the other hand, is known to protect against the development of atherosclerosis and is inversely correlated with the incidence of CVD.^[^ ^4^, ^5^, ^6^ ^]^ There is a growing body of literature to suggest that the function of HDL is more important than the circulating levels of HDL in protecting against CVD.^[^ ^7^ ^]^ Indeed, paraoxonase 1 (PON1), an alipoprotein A1 (Apo‐A1) coordinating enzyme that is almost exclusively located on HDL, has the capacity to protect LDL from oxidative modifications^[^ ^8^ ^]^ by free radicals that are produced by endothelial cells thus inhibiting atherosclerotic development. Polymorphisms in the PON1 gene, in particular the Q192R and L55M single nucleotide polymorphisms (SNPs), are associated with lipoprotein oxidation and individuals with the 192 Q/Q and 55 M/M PON1 genotypes gain greater protection from CVD.^[^ ^9^ ^]^

Anthocyanins are natural plant pigments that exist in a variety of berry fruits and other foods such as aubergine skins, black rice, red/purple potatoes, cabbage, and grapes. Anthocyanins are glycosides of anthocyanidins and are the natural form of these flavonoids in plants. There are a number of reported dietary interventions that provide evidence that dietary supplementation with berry fruits, berry fruit extracts, and purified anthocyanin mixtures can increase HDL‐C,^[^ ^10^, ^11^, ^12^, ^13^, ^14^, ^15^ ^]^ reduce LDL‐C,^[^ ^12^, ^14^, ^16^, ^17^ ^]^ and triglycerides^[^ ^10^, ^14^ ^]^ and enhance HDL‐associated PON1 activity^[^ ^18^ ^]^ in both healthy individuals and those with hypercholesterolemia. However, the data are equivocal, and some studies have not observed significant effects on these biomarkers. For example, in a systematic review of randomized controlled trials assessing the effects of anthocyanin‐rich foods, anthocyanin‐rich crude extracts or highly purified mixed anthocyanin supplements on biomarkers of cardiovascular disease risk, the authors noted that some studies reported significant improvements in lipoprotein profiles while others reported a lack of effects. However, the authors noted that all of the four studies that were conducted with hypercholesteremic individuals (total cholesterol >5.0 mmol L^−1^) reported significant (and substantial) decreases in plasma LDL‐C,^[^ ^19^ ^]^ a finding which was confirmed in two more recently published systematic reviews. These studies were performed with relatively high doses of anthocyanins (90 or 320 mg day^−1^) in different populations, two in Iran^[^ ^10^, ^11^ ^]^ and two in China.^[^ ^16^, ^17^ ^]^ These observations support the notion that mixtures of anthocyanins are effective at improving lipoprotein profiles in people with elevated cholesterol which signifies dyslipidemia but may be less effective in people with normal cholesterol levels.

There are three main types of anthocyanins based on the number of hydroxyl groups on the B‐ring of the anthocyanidin moiety: pelargonidin‐type (4’‐monohydroxyl), cyanidin‐type (3’,4’‐dihydroxyl), and delphinidin‐type (3’,4’5’‐trihydroxyl). Although there are numerous substitutions (e.g., glycosylation, methylation, and acylation of sugar substituents) that give rise to a much larger number of structures, all anthocyanins are primarily derived from these three structures. The major anthocyanins in human diets are the cyanidin‐type and delphinidin‐type. Bilberries are reported to contain ∼285 mg anthocyanins per 100 g fresh fruit and the majority of the anthocyanins are of the delphinidin‐type.^[^ ^20^ ^]^ Black rice is a small group of anthocyanin‐rich varieties of rice (*Oryza sativa*) that are reported to contain ∼300 mg anthocyanins 100 g^−1[^ ^21^ ^]^ and contains almost exclusively cyanidin‐type anthocyanins.^[^ ^22^ ^]^ The relationship between anthocyanin structure and biological activity is not well understood but there is evidence to show that is does influence both absorption (pelargonidin > cyanidin > delphinidin)^[^ ^23^ ^]^ and subsequent bioactivity.^[^ ^24^ ^]^

Since there have been no studies reported to date that have tested the efficacy of the main types of anthocyanins in isolation (e.g., cyanidin‐type vs delphinidin‐type), the aim of this study was to i) determine the effects of these two major dietary types of anthocyanins on LDL‐C and other cardiometabolic markers for CVD risk in hyperlipidaemic individuals and ii) explore possible relationships between the effects of the two anthocyanin types on cardiometabolic markers for CVD risk and PON1 genotype.

## 2 Experimental Section

### 2.1 Study Population

Fifty‐two, apparently healthy men and women aged 45+ years with a total cholesterol conc. ≥5 mmol L^−1^ at eligibility assessment were recruited within Norwich, UK. The study exclusion criteria were: current or recent smoker; medical conditions judged to affect the study outcome or compromise the well‐being of the participant (e.g., gastrointestinal disease, diabetes, and cancer); medications judged to affect the study outcome (e.g., lipid lowering therapy); some dietary supplements (anthocyanin and other polyphenol‐rich botanicals); food products known to reduce cholesterol (e.g., Benecol, Flora ProActiv); clinical results at eligibility assessment judged to affect the trial outcome or be indicative of a health problem.

The study period was from November 2017 to September 2018. The study was conducted in the Human Nutrition Unit at the Quadram Institute Bioscience (QIB), Norwich, UK and all procedures were approved by both the QIB Human Research Governance Committee and the West Midlands – South Birmingham Research Ethics Committee (ref no: 17/WM/0154). Each participant gave written informed consent prior to taking part in the study. The study was registered with clinicaltrials.gov (Ref: NCT03213288).

### 2.2 Study Design

The study was a randomized, placebo‐controlled, double‐blind, three arm crossover design investigating the effects of repeated daily consumption of black rice and bilberry fruit derived anthocyanins on cardiometabolic markers for CVD. The three treatments were: i) a bilberry extract delivering 320 mg of mostly delphinidin/trihydroxy type anthocyanins (BE), ii) a black rice extract delivering 320 mg of mostly cyanidin/dihydroxy type anthocyanins (BRE), and iii) a placebo control. Both extracts and placebo were all delivered in the same opaque, cellulose based capsules that were suitable for oral consumption (K‐caps vegetarian capsules; GoCaps).

The primary outcome measure for this study was LDL‐C concentration. Secondary outcome measures were concentrations of other blood lipids and lipoproteins (total and HDL‐C, triglycerides, ApoA1, ApoB1, HDL3), bile acids, biomarkers of glycemic control (glucose and fructosamine), and biomarkers of PON1 status.

The BE, BRE, and placebo were encapsulated, and the capsules were bottled before the start of the study. The capsules were identical in appearance after filling and cleaning. Blinding of the study was achieved by designating the treatments and labeling bottles with a letter, A, B, or C, by a person not assigned to the study. The order in which the participants ingested the treatments was determined by a computer generated (randomisation.com) sequence of letters from A to C using a block randomization approach. Participants meeting the eligibility criteria were allocated sequentially to a treatment sequence upon enrollment, by the study manager. The principal investigator, study manager, study nurse, participants, and statistician were blinded to the treatments and un‐blinding was only done upon completion of the statistical analyses. The treatments were relabeled prior to statistical analysis so that the statistician received the data for each participant with an allocation code for each arm/treatment, but the statistician was not aware of which allocation code corresponded with which treatment or control.

For each of the treatments, participants were asked to consume the capsules (*n* = 4) once daily, in the morning, for 28 days with a minimum 2‐week washout period between arms. To aid compliance, participants were provided with a capsule checklist and asked to make a tick on the check list each day the capsules were ingested. Compliance was assessed from the checklist and by counting the unused capsules returned at the end of the 4‐week treatment period.

Participants were required to restrict their berry fruit intake to a combined maximum of three portions per week. The restrictions started 2 weeks before commencing the first treatment period and continued until completion of the study, including wash‐out periods. In addition, berry fruits were completely excluded from the diet for the 24 h period preceding the pre and post treatment assessment days. A list of prohibited fruits was provided to the participants to aid compliance.

All outcome measures were assessed at the start and end of each 28‐day treatment period. To investigate the chronic effects of anthocyanins on the previously described cardiometabolic risk markers for CVD, measurements were made on day 1 (baseline) and day 29 of each treatment period.

### 2.3 Dosage Information

Participants were asked to consume a total of 320 mg of purified anthocyanins per day for 28 days. The anthocyanins (black rice extract, bilberry extract) were purchased from the Beijing Ginko Group (BGG, China) and were analyzed for their content and composition of anthocyanins and other phenolic components and were shown to be highly purified anthocyanin powders (see following section). Each extract was separately mixed with a pre‐determined proportion of microcrystalline cellulose so that the capsule filling process generated capsules with the correct dose (±1%) of anthocyanins. The dose of 320 mg total anthocyanins per day was chosen because i) this dose had been used in dietary intervention studies with anthocyanins that had been reported to have cholesterol lowering effects,^[^ ^16^, ^17^ ^]^ and ii) this dose was achievable in a normal diet (e.g., 100 g of dry weight black rice, fresh weight bilberries, and fresh weight blackcurrants would be expected to contain about 300,^[^ ^21^ ^]^ 285,^[^ ^20^ ^]^ and 595 (www.phenol‐explorer.eu) mg of total anthocyanins, respectively).

### 2.4 Analysis of Bilberry and Black Rice Extracts

Both extracts were analyzed in‐house to confirm anthocyanin content and to determine the correct mass of active material required to deliver the 320 mg daily dose of anthocyanins. In brief, extracts were analyzed for anthocyanins by reverse phase HPLC/MS (Agilent) using a Kinetex XB‐C18 column (100 × 4.6 mm; 2.6 µM). The mobile phase consisted of 5% aqueous formic acid (solvent A) and 5% formic acid in acetonitrile (solvent B). Samples were eluted with the use of a gradient of increasing solvent B from solvent A as follows: 5% B at 0 min, 7% B at 10 min, 10% B at 15 min, 13% B at 16:50 min, 20% B at 18 min, 5% B at 20–25 min. The flow rate was 1 mL min^−1^. The anthocyanins were detected by diode array detection (DAD) and the detection was established at 520 nm. The anthocyanin composition of the BE and BRE was shown in **Table** **1**. In addition, the extracts were analyzed for a range of simple phenolics and flavonoids using a modified version of the above gradient on the same column and a DAD monitoring across 200–700 nm, and for pro(antho)cyanidins using a previously published method based on HILIC chromatography with fluorescence detection.^[^ ^25^ ^]^ No proanthocyanidins were detected and only very low concentrations of a few phenolics were quantifiable, but with the total accounting for <1% of the total phenolics including anthocyanins in the extracts.

**Table 1:** Composition (mg) of bilberry and black rice extracts according to anthocyanin type and hydroxyl group (structure)

Anthocyanin composition	Bilberry extract	Black rice extract
*Anthocyanin type*		
Delphinidin	135.8	0.0
Cyanidin	54.0	297.8
Peonidin	14.9	22.2
Petunidin	82.5	0.0
Malvidin	32.9	0.0
Total	320.0	320.0
*Anthocyanin structure*		
Tri‐hydroxyl	251.1	0.0
Di‐hydroxyl	68.9	320.0
Total	320.0	320.0

Bilberry extract anthocyanins were 3‐O‐galactosides/‐glucosides/‐arabinosides of delphinidin, cyanidin, peonidin, petunidin, and malvidin; Black rice extract anthocyanins were 3‐O‐glucoside (89.3%), 3,5‐O‐diglucoside (1.2%), and 3‐O‐(6“‐O‐*p*‐coumaryl) (1.4%) of cyanidin and 3‐O‐glucoside (7.2%) and 3‐O‐(6”‐O‐*p*‐coumaryl) (1.9%) of peonidin.

### 2.5 Analysis of Cardiometabolic Markers for CVD

Whole blood was collected into EDTA and serum separating tubes (Becton‐Dickenson). Samples collected into EDTA tubes were immediately centrifuged at 2500 x *g* for 10 min. Samples collected in serum separating tubes were centrifuged after 30 min at 2000 x *g* for 10 min. Post centrifugation, plasma, and serum were sub‐aliquoted into appropriate collection tubes, snap frozen, and then stored at −80 °C until analysis.

Serum lipids (total/HDL/LDL‐C, triglycerides) and biomarkers of HDL function (ApoA1 ApoB, HDL3) and glycemic control (glucose, fructosamine) were analyzed with enzymatic reagents using an automated clinical chemistry analyzer (Randox; Daytona plus), according to the manufacturer's instructions.

PON1 arylesterase and lactonase activity was measured in blood serum by monitoring the absorbance at wavelengths 410 and 412 nm respectively, using a FLUROstar Optima microplate reader (BMG, Labtech, UK). To ensure that the study was only quantifying the enzyme activities mediated by PON1 (and not other serum arylesterases/lactonases), PON1 lactonase/arylesterase was measured with and without the addition of 100 µM of 2‐hydroxyquinoline (2‐HQ); a selective competitive inhibitor of PON. PON1‐mediated arylesterase/lactonase activity was then calculated by the subtraction of activities in the presence of 2‐HQ from the total arylesterase/lactonase in the absence of 2‐HQ.

### 2.6 Extraction and Analysis of Bile Acids

Plasma (500 µL) was added to 70% methanol (1.0 mL) and d4‐DCA (25µl; 40 µg mL^−1^) and vortexed for 60 s. The precipitated protein was then centrifuged (3000 rpm; 4 °C; 10 min) and the supernatant transferred to a tube containing d4‐CDCA (25 µL; 40 µg mL^−1^). The sample was then passed through a hydrophilic‐lipophilic balance clean‐up cartridge (Waters Oasis Prime HLB, 1 cm^3^, 30 mg), washed with 5% methanol (1.0 mL) and eluted in methanol (500 µL) before addition of d4‐GCA and d4‐LCA (25 µL; 40 µg mL^−1^). Of the internal standards added, d4‐GCA was the primary reference internal standard with the others monitored as checks in the extraction procedure.

In brief, extracts were analyzed using HPLC (Agilent 1260) coupled to a triple quadrupole mass spectrometer (AB Sciex; 4000 QTrap) using a Supelco Ascentis Express C18 column (150 × 4.6 mm; 2.7 µM) maintained at 40 °C. The mobile phase consisted of water, 5mM ammonium acetate and 0.012% formic acid (solvent A) and methanol, 5mM ammonium acetate and 0.012% formic acid (solvent B) at a flow rate of 600 µL min^−1^. Injection was made at 50% B and held for 2 min, increased to 95% B at 20 min and held until 24 min. The mass spectrometer was operated in electrospray negative mode with capillary voltage of −4500 V at 550 °C. Instrument specific gas flow rates were 25 mL min^−1^ curtain gas, GS1: 40 mL min^−1^ and GS2: 50 mL min^−1^. Dwell time for each MRM was 20 ms. Quantification of the analytes was applied using Analyst 1.6.2 software to integrate detected peak areas relative to the deuterated internal standards.

### 2.7 PON1 Genotyping

All participants were genotyped for their Q192R and L55M polymorphisms using PCR reactions with primers designed to amplify the exons that contain the SNPs. The amplicons were sequenced using standard sequencing techniques. The sequences were then aligned to the reference genome to identify the genotype. DNA was extracted and purified from whole blood using a DNeasy Blood & Tissue Kit (Qiagen, Cat# 69 504) according to the manufacturer's protocol. PCR reactions were performed with two different sets of primers that were designed specifically to amplify exon 3 (which contained the L55M polymorphism) and exon 6 (which contained the Q192R polymorphism) using a method described elsewhere.^[^ ^26^ ^]^ PCR products were purified using a QIAquick PCR purification kit (Qiagen, Cat# 28 104) according to the manufacturer's protocol. The purified products were sent for sequencing (Eurofins, UK). To determine the genotypes, the sequences were aligned to the human reference genome and SNPs were identified using Ugene software for windows version 1.29.0.

### 2.8 Extraction and Quantification of Anthocyanin Metabolites in Urine

Participants collected all urine in the 24 h period prior to the first day of each treatment and for the 24 h period at the end of each treatment to the day 29 morning when blood samples were taken. They were provided with 2.5 L plastic urine collection bottles to which 1 g of ascorbic acid had been previously added as a preservative. Urine samples (1.0 mL) were subjected to solid phase extraction (SPE) using pre‐conditioned Strata‐X cartridges and then evaporated to approximately 200 µL volume. Evaporated extracts (100 µL) were then spiked with D9‐caffeine (200 µM; 10 µL) as a volume control marker. Extracts were further diluted with 1% formic acid in water (100 µL). Extracts were analyzed using LC‐MS/MS (Agilent 6490) using multiple reaction monitoring operated in positive mode and an ACQUITY UPLC HSS T3 column (2.1 × 100 mm; 1.8 µm). The mobile phase consisted of 5% formic acid in water (solvent A) and 5% formic acid in acetonitrile (solvent B). Extracts were eluted with the use of a gradient of increasing solvent B from solvent A as follows: 5% B at 1 min, 10% B at 5 min, 25% B at 30 min, 95% B at 40 min, 95% B at 41.1 min, and 5% B at 46 min. The flow rate was 0.4 mL min^−1^.

### 2.9 Statistical Analysis

The primary outcome measure for this study was LDL‐C. The study was powered to detect a change of 0.14 mmol L^−1^ in LDL‐C (relative to placebo) at a significance level of 0.05 with 90% power. Assuming a standard deviation similar to that observed in the data from Zhu et al.^[^ ^17^ ^]^ (approximately 0.5 mmol L^−1^), the calculation supported the recruitment of 50 participants in total.

All outcomes were analyzed independently using linear mixed models. Effects were estimated by the interaction between treatment and time (pre‐ vs post treatment). The effect of treatment order and its interaction with time were also included in each model along with a single random effect of participant. All models were estimated on the natural scale for ease of interpretation, and on the logarithmic scale as a sensitivity analysis to correct heteroskedasticity that was evident for some outcomes. There was no difference in interpretation of results under either model.

## 3 Results

### 3.1 Study Population

**Figure 1:**
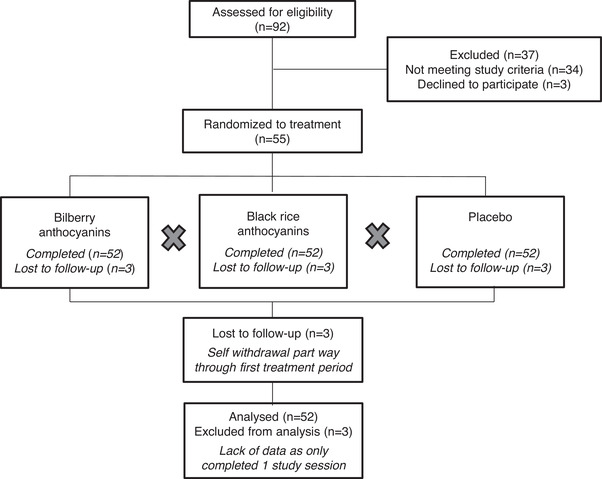
Flow of participants through the study.

Fifty five participants were randomly assigned to a treatment sequence. Three participants self‐withdrew part way through the first treatment period. Difficulties with study time commitments were given as the reason for withdrawal. Therefore, 52 participants completed the study (24 men and 28 women). No serious adverse events were reported during the study. **Table** **2** shows the participant characteristics and biomarkers of CVD risk at the start of trial and prior to any treatments. **Figure** **1** shows the flow of participants throughout the study.

**Table 2:** Participant characteristics and biomarkers of CVD risk at baseline

Characteristic	Men	Women	All
Age [yr]	63.1 ± 8.4	62.2 ± 7.4	62.6 ± 7.8
BMI [kg m^−2^]	25.7 ± 2.6	26.1 ± 4.0	25.9 ± 3.4
Total cholesterol [mmol L^−1^]	5.7 ± 0.8	6.2 ± 1.0	5.9 ± 0.9
LDL cholesterol [mmol L^−1^]	3.9 ± 0.8	3.9 ± 0.9	3.9 ± 0.8
HDL cholesterol [mmol L^−1^]	1.6 ± 0.4	2.0 ± 0.5	1.8 ± 0.5
Triglycerides [mmol L^−1^]	1.3 ± 0.5	1.2 ± 0.6	1.2 ± 0.6
HDL3 [mg dL^−1^]	26.6 ± 4.0	28.2 ± 3.7	27.5 ± 3.9
ApoA1 [mg dL^−1^]	212 ± 43	244 ± 73	229 ± 63
ApoB [mg dL^−1^]	126 ± 23	129.7 ± 28.1	128.0 ± 25.7
PON1 aryl [µmol min^−1^ mL^−1^]	3.5 ± 1.1	4.0 ± 2.0	3.8 ± 1.7
PON1 lact [µmol min^−1^ mL^−1^]	6.4 ± 1.2	6.8 ± 2.1	6.6 ± 1.8
Fructosamine [µmol L^−1^]	268 ± 17	270 ± 26.0	269 ± 22
Glucose [mmol L^−1^]	5.7 ± 0.4	5.3 ± 0.4	5.5 ± 0.4
Total bile acids [ng mL^−1^]	1333** **± 550	1105** **± 893	1210** **± 756

Values are means ± sd; *n* = 52 participants (24 men and 28 women).

Overall compliance to treatments was high. When assessed as a proportion of the intended total, >99% of capsules were ingested across all three treatments.

Only data from the 52 participants who completed the trial are reported. Of these 52, a total of two observations in two outcomes were missing, otherwise all other data for the 52 participants is included.

### 3.2 Excretion of Anthocyanin Metabolites in Urine

In the baseline 24‐h urine collections, very small quantities of total anthocyanins were detected in all participants, but the amount detected was similar across all three treatment arms (28 ± 36, 23 ± 25, and 30 ± 62 nmols; placebo, BRE, and BE, respectively). After 28‐days ingestion of the BRE and BE, the amount of anthocyanins detected in the 24‐h urine collections was substantially higher (631 ± 539 and 299 ± 204 nmols; BRE and BE, respectively), indicating excellent compliance with the capsule ingestion regime.

### 3.3 Effects of Treatments on Cardiometabolic Markers of CVD

The mean values in cardiometabolic risk markers before and after treatment with the BE, BRE, and placebo are shown in **Table** **3**. After 4 weeks ingestion of BE and BRE, no significant differences were observed in any of the assessed biomarkers of vascular function (total/HDL/LDL‐C, triglycerides, and ApoB), glycemic control (glucose, fructosamine), HDL function (ApoA1, HDL3, PON1 arylesterase, and lactonase activity), or plasma bile acids, compared with placebo.

**Table 3:** Changes in biomarkers of CVD risk 28‐days after ingestion of bilberry extract, black rice extract, and placebo

Variable	Placebo		Black rice extract				Bilberry extract			
	Pre	Post	Pre	Post	Change	*p* ^b)^	Pre	Post	Change^b)^	*p*
Total cholesterol [mmol L^−1^]	6.0 ± 0.9	5.9 ± 1.0	5.9 ± 1.0	5.9 ± 1.0	0.04 (−0.15–0.23)	0.72	6.0 ± 1.0	6.0 ± 1.0	0.02 (−0.17–0.21)	0.83
LDL cholesterol [mmol L^−1^]	3.9 ± 0.8	3.8 ± 0.9	3.9 ± 0.8	3.9 ± 0.8	0.08 (−0.08–0.24)	0.36	3.9 ± 0.8	3.9 ± 0.8	−0.00 (−0.16–0.16)	0.99
HDL cholesterol [mmol L^−1^]	1.8 ± 0.5	1.9 ± 0.5	1.8 ± 0.5	1.8 ± 0.5	0.01 (−0.07–0.09)	0.79	1.8 ± 0.5	1.8 ± 0.5	−0.02 (−0.10–0.06)	0.65
Triglycerides [mmol L^−1^]	1.2 ± 0.6^a)^	1.2 ± 0.6	1.2 ± 0.6	1.2 ± 0.6	–0.01 (0.14–0.13)	0.94	1.2 ± 0.6	1.3 ± 0.6	0.08 (–0.06–0.21)	0.27
HDL3 [mg dL^−1^]	27.5 ± 4.4	27.8 ± 4.3	27.5 ± 4.0	27.6 ± 4.1	0.08 (‐0.88 ‐ 1.04)	0.87	27.5 ± 4.4	27.8 ± 4.3	0.27 (‐0.69 ‐ 1.23)	0.59
ApoA1 [mg dL^−1^]	228.4 ± 62.0	227.6 ± 60.3^3^	225.8 ± 60.2	225.9 ± 57.0	1.27 (–4.85–7.39)	0.69	224.8 ± 56.0	227.6 ± 60.3	4.00 (–2.12–10.12)	0.21
ApoB [mg dL^−1^]	128.9 ± 27.5	127.2 ± 27.4	128.2 ± 26.9	128.0 ± 26.6	1.53 (–2.62–5.69)	0.48	128.1 ± 24.6	127.6 ± 27.3	1.14 (–3.02–5.30)	0.59
PON1 Aryl [µmol min^−1^ mL^−1^]	3.7 ± 1.6	3.8 ± 1.6	3.8 ± 1.7	3.8 ± 1.7	−0.12 (−0.26–0.02)	0.09	3.7 ± 1.6	3.8 ± 1.6	−0.01 (−0.16–0.13)	0.86
PON1 Lact, [µmol min^−1^ mL^−1^]	6.7 ± 1.7	6.6 ± 1.7	6.5 ± 1.8	6.6 ± 1.8	0.08 (−0.12–0.27)	0.43	6.6 ± 1.8	6.7 ± 1.8	0.14 (−0.06–0.33)	0.17
Glucose [mmol L^−1^]	5.5 ± 0.4	5.5 ± 0.4	5.5 ± 0.5	5.5 ± 0.5	0.01 (−0.12–0.15)	0.84	5.5 ± 0.4	5.4 ± 0.4	−0.07 (−0.20–0.07)	0.33
Fructosamine [µmol L^−1^]	266.4 ± 25.6	65.8 ± 23.7	265.2 ± 23.0	265.7 ± 24.4	1.06 (−3.56–5.67)	0.66	266.0 ± 24.6	265.7 ± 24.5	0.42 (−4.19–5.04)	0.86
Total bile acids [ng mL^−1^]	1122 ± 567	1351 ± 1000	1157 ± 749	1251 ± 841	−134 (−483–216)	0.46	1230 ± 823^3^	1214.7 ± 699.2	−243.4 (−593.9–107.2)	0.81

^a)^ Values are mean ± SD; change = post minus pre‐treatment versus post minus pre placebo with 95% CI and *p*‐values

^b)^ *n* = 51 participants (all others *n* = 52). Data were analyzed independently using linear mixed models. Effects were estimated by the interaction between treatment and time (pre‐vs post treatment).

### 3.4 Association between Treatments, PON1 Genotype and Biomarkers of CVD

Participants were categorized according to their Q192R and L55M genotype and the interaction between treatments, genotype, and biomarkers of CVD investigated. No significant changes in HDL function (**Table** **4**) or other biomarkers of CVD risk (data not shown) were observed after ingestion of BRE and BE derived anthocyanins regardless of genotype.

**Table 4:** Association between biomarkers of HDL function, genotype and ingestion of bilberry extract, black rice extract, and placebo

	Q192R			v		
	QQ	QR	RR	LL	LM	MM
*Placebo*						
HDL [mmol L^−1^]	0.03 (0.05)	0.00 (0.04)	0.07 (0.09)	0.07 (0.05)	−0.01 (0.04)	−0.05 (0.10)
HDL3 [mg dL^−1^]	0.55 (0.54)	–0.46 (0.51)	–0.03 (1.03)	0.49 (0.54)	–0.45 (0.51)	0.27 (1.14)
ApoA1 [mg dL^−1^]	−0.14 (3.51)	−1.81 (3.32)	−2.19 (6.64)	−1.82 (3.50)	−0.51 (3.29)	−1.19 (7.88)
PON1 Aryl [µmol min^−1^ min^−1^]	0.11 (0.08)^a)^	0.15 (0.08)	−0.09 (0.15)	0.13 (0.08)	0.07 (0.08)	0.14 (0.17)
PON1 Lact [µmol min^−1^ min^−1^]	−0.06 (0.11)	0.00 (0.10)	−0.25 (0.21)	0.03 (0.11)	−0.09 (0.10)	−0.25 (0.23)
*Black rice extract*						
HDL [mmol L^−1^]	0.04 (0.05)	0.03 (0.04)	0.05 (0.09)	0.06 (0.05)	0.03 (0.04)	−0.06 (0.10)
HDL3 [mg dL^−1^]	−0.03 (0.54)	0.32 (0.51)	−0.29 (1.03)	0.34 (0.54)	−0.07 (0.51)	−0.05 (1.14)
ApoA1 [mg dL^−1^]	2.23 (3.47)	−0.86 (3.32)	−3.43 (6.64)	−0.57 (3.50)	0.84 (3.29)	−0.15 (7.35)
PON1 Aryl [µmol min^−1^ min^−1^]	0.00 (0.08)	−0.05 (0.08)	0.08 (0.15)	0.04 (0.08)	−0.04 (0.08)	−0.15 (0.17)
PON1 Lact [µmol min^−1^ min^−1^]	0.13 (0.11)	−0.06 (0.10)	−0.01 (0.21)	0.09 (0.11)	−0.01 (0.10)	−0.09 (0.23)
*Bilberry extract*						
HDL [mmol L^−1^]	−0.02 (0.05)	0.02 (0.04)	0.03 (0.09)	−0.01 (0.05)	0.00 (0.04)	0.03 (0.10)
HDL3 [mg dL^−1^]	0.01 (0.54)	0.57 (0.51)	0.16 (1.03)	0.13 (0.54)	0.43 (0.51)	0.20 (1.14)
ApoA1 [mg dL^−1^]	3.32 (3.47)	2.91 (3.32)	0.34 (6.64)	3.86 (3.50)	1.24 (3.29)	5.83 (7.35)
PON1 Aryl, [µmol min^−1^ min^−1^]	0.07 (0.08)	0.11 (0.08)	0.07 (0.15)	0.02 (0.08)	0.16 (0.08)	0.05 (0.17)
PON1 Lact, [µmol min^−1^ min^−1^]	0.03 (0.11)	0.13 (0.10)	0.03 (0.21)	−0.03 (0.11)	0.16 (0.10)	0.10 (0.23)

^a)^ Change = post minus pre‐values for placebo, black rice extract and bilberry extract.

## 4 Discussion

The main finding of this study was that ingestion of 320 mg day^−1^ of delphinidin or cyanidin type anthocyanins for 28‐day did not reduce LDL‐C in a study population with elevated cholesterol levels. Neither did consumption of delphinidin or cyanidin type anthocyanins beneficially alter other biomarkers related to vascular function (total cholesterol, HDL‐C, triglycerides, and ApoB) and glycemic control (glucose, fructosamine) or biomarkers of HDL function (ApoA1, HDL3 and PON1 arylesterase, and lactonase activity). The collected evidence supports that compliance with the dietary intervention was excellent, and therefore there are other reasons for the lack of observed effects.

Several human intervention trials investigating the effects of anthocyanins on biomarkers of CVD risk, have reported increases in HDL‐C and reductions in LDL‐C and triglycerides after ingestion. For example, in a recent systematic review and meta‐analysis of 17 RCTs^[^ ^27^ ^]^ assessing the impact of crude or purified anthocyanins on inflammatory markers and lipid profile in both healthy and “at risk” individuals, 15 studies reported on the changes in lipid markers associated with CVD. Of these 15 studies, several reported statistically significant increases in HDL‐C^[^ ^11^, ^16^, ^17^, ^28^, ^29^, ^30^ ^]^ and reductions in LDL‐C^[^ ^10^, ^11^, ^16^, ^17^, ^28^, ^29^, ^30^, ^31^ ^]^ and three reported statistically significant reductions in total cholesterol and triglycerides.^[^ ^10^, ^11^, ^27^ ^]^ When comparing the similarities across the studies included in this meta‐analysis, six^[^ ^16^, ^17^, ^28^, ^29^, ^30^, ^31^ ^]^ of the eight studies were conducted using conditions that were in keeping with that of our study, e.g., participants were ≥40 years of age, hypercholesterolemic prior to starting the treatments (mean total cholesterol and LDL‐C across the six studies was 6.07 and 3.6 mmol L^−1^, respectively) and ingested an anthocyanin dose of 320 mg per day. The duration in which participants ingested the anthocyanins in these six studies ranged from 4 weeks^[^ ^10^ ^]^ to 12 and 24 weeks in the other five reports. There is therefore evidence of effects on LDL‐C being observed in a dietary intervention of 4 weeks, but the majority of studies reporting lowered cholesterol were longer in duration. It is interesting to note that another recent systematic review and meta‐analysis of 19 RCTs^[^ ^32^ ^]^ on the effects of anthocyanins on biomarkers of CVD in which the authors reported that pooled results of all 19 RCTs showed no significant effects of anthocyanin supplementation on increasing HDL‐C and reducing LDL‐C (which is in keeping with our study), the authors concluded that after sub‐group analysis by dose and duration of exposure, anthocyanins were effective at reducing total and LDL‐C at doses ≥300 mg day^−1^ for more than 12 weeks duration. However, the fact that the majority of studies were of longer duration may have biased this analysis, and the effect size reported after 4 weeks consumption of 90 mg anthocyanins from *Vaccinium arctostaphylos* was large (−11.8 mg dL^−1^, –9.8%) and highly significant (*p* = 0.004).

Improving HDL functionality is considered to be an important tool for ameliorating the risk of developing CVD. ApoA1 and PON1 are HDL associated proteins known to have an atheroprotective role. In recent years, PON1 has been the focus of much research activity because of its ability to protect HDL and LDL from oxidative modifications, which in turn may inhibit the atherosclerotic process underlying CVD. Despite the relative importance of PON1 there are few published studies reporting on the effects of flavonoids on PON1 activity and to our knowledge, only one study reports on the effects of purified anthocyanins. After feeding hypercholesterolemic adults 320 mg day^−1^ of purified anthocyanins for a period of 24 weeks, Zhu et al.^[^ ^18^ ^]^ report that PON1 activity was significantly increased by 17%. This is in direct contrast to the findings from the study reported here. There are two possible reasons for the differences. Firstly, anthocyanin exposure in the study conducted by Zhu et al. was six times longer than that of our study. Secondly, we measured PON1 mediated activity using a potent inhibitor that eliminated other interfering arylesterases and lactonases. It is possible that the presence of other interfering enzymes could have resulted in an over‐estimate of the treatment effect on PON1 activity reported in the study conducted by Zhu et al.

It is well established that polymorphisms in the PON1 gene affect PON1 enzyme activity^[^ ^33^, ^34^ ^]^ and the PON1 Q192R and L55M in particular are well studied polymorphisms because they are associated with CVD. However, few studies have investigated the interaction between anthocyanin ingestion, biomarkers of CVD and PON1 genotype. Rizzi et al.^[^ ^35^ ^]^ studied the interaction between polyphenol intake (including anthocyanins) and PON1 variants on the lipid profiles of 443 healthy participants using an observational nutrigenetic approach. In this study, the authors report a significant association between increased HDL levels in those individuals consuming high amounts of anthocyanins in four PON1 polymorphisms. Regardless, no significant interaction between the anthocyanin treatments and biomarkers of CVD and PON1 genotype was observed in our study, possibly because the population size was too small.

In the study reported here, we did not observe a beneficial effect of anthocyanins on measures of glycemic response. Of the six aforementioned studies reporting the effects of 320 mg day^−1^ of purified anthocyanins on biomarkers for CVD risk, four included fasting plasma glucose as an outcome measure for statistical evaluation.^[^ ^16^, ^29^, ^30^, ^31^ ^]^ Our findings are in keeping with three of these studies^[^ ^16^, ^30^, ^31^ ^]^ in which the authors report no significant reduction in plasma glucose levels after 12 weeks anthocyanin supplementation. While the fourth study did report a borderline statistically significant reduction in fasting plasma glucose (*p* = 0.042), the study was conducted in a population of type 2 diabetics with a baseline plasma glucose concentration above optimal (7.1 mmol L^−1^) and a supplementation period of 24 weeks.^[^ ^29^ ^]^

The main strength of our study was the use of highly purified preparations of two structurally different anthocyanins (trihydroxyl and dihydroxyl; delphinidin and cyanidin type anthocyanins, respectively), thus providing meaningful information regarding the effects of anthocyanin structure on biological activity. The anthocyanins were highly purified which meant that the data we generated allowed us to come to conclusions about the specific ability of the anthocyanins themselves to cause changes in the measured biomarkers. Use of isolated anthocyanins also eliminated other dietary components that could have negatively impacted on the absorption and metabolism of the anthocyanins. Another strength was the well powered cross‐over design approach with each outcome measured before and after each treatment which helps reduce the effects of biologically relevant confounding variables when assessing the effects of a treatment. Using a cross‐over design allowed us to include a greater number of participants in each of the treatment and control groups. If we had used a parallel design, we would have needed about four times as many participants to achieve similar power. In addition, the study population were hypercholesterolemic, and by virtue more likely to be susceptible to beneficial changes of a treatment. Taking these factors into account we are able to rule out any clinically significant effect of structurally different bilberry and black rice derived anthocyanins, when ingested for 4 weeks, on any of the biomarkers for CVD that we measured in this study population.

Our study was not without its limitations. The “trade off” between a cross over design approach (that minimizes biological variation between participants) and a parallel design approach (that offers the potential for a longer exposure period to a treatment) is an important consideration for any researcher when designing a human intervention trial. We chose the former and this may have been the reason for not seeing the effect observed in other studies. In addition, the use of highly purified anthocyanins rather than a whole food (black rice grains, bilberry fruits) did not allow our study to observe possible effects induced by a complex mixture of anthocyanins with macronutrients, micronutrients, and other phytochemicals.

In conclusion, our data show that ingestion of 320 mg day^−1^ of bilberry‐derived anthocyanins or black rice‐derived anthocyanins for 28 days did not reduce LDL‐C. Neither did it improve biomarkers of HDL function, glycemic control or PON1 arylesterase and lactonase activity. The lack of effects may be due to the short duration of the treatments. Conducting studies over longer time periods (≥12 weeks duration) should be considered in future research.

## Conflict of Interest

The authors declare no conflict of interest.

## Author Contributions

P.A.K., W.J.H., and H.A., designed the study; W.J.H. was responsible for participant recruitment and day‐to‐day management of the trial; H.A., J.P., and M.P. analyzed biological samples; G.M.S. analyzed the study data; W.J.H. and P.A.K. wrote the manuscript with contributions from all other authors; P.A.K. had primary responsibility for final content. All authors read and approved the final manuscript.

## Data Availability

The data that support the findings of this study are available from the corresponding author upon reasonable request.
